# Response to “radiomics of mandibular osteoradionecrosis with recurrence: The need for etiologic and temporal validation”

**DOI:** 10.1016/j.phro.2026.101108

**Published:** 2026-10-22

**Authors:** Arnaud Beddok

**Affiliations:** a Department of Radiation Oncology, Institut Godinot, Reims, France; b Université de Reims Champagne-Ardenne, CRESTIC EA, Reims, 3804, France

**Keywords:** Osteoradionecrosis, Radiomics, Head and neck cancer, Computed tomography, Recurrence

Dear Editor,

I thank Topkan and colleagues for their thoughtful comments on our study [1] and welcome the opportunity to clarify its scope and interpretation. Their correspondence raises important considerations regarding the etiological characterization of osteoradionecrosis (ORN), the temporal relationship between imaging and pathological assessment, and the interpretation of exploratory radiomic findings.

First, I agree that histopathology alone cannot establish the radiation-induced etiology of mandibular osteonecrosis. However, this was not how ORN was diagnosed in our cohort. All included patients had been clinically and radiologically diagnosed with mandibular ORN in the context of previous head-and-neck radiotherapy and underwent surgery for this condition. Histopathological examination of the surgical specimen was subsequently used to confirm necrosis and, critically for our study objective, to determine the presence or absence of associated tumor recurrence. Thus, the term “pathologically confirmed ORN” should not be interpreted as indicating that radiation causation was established by histopathology alone. This approach is consistent with contemporary definitions integrating previous radiation exposure with clinical and radiological findings [2], [3]. Nevertheless, I agree that site-specific mandibular dosimetry and systematic documentation of potential competing etiologies would further strengthen etiological characterization in future studies.

Second, Topkan et al. appropriately highlight the interval between the CT used for radiomic analysis and surgery (median, 86 days; IQR, 45–200) [1]. Surgical pathology establishes recurrence at surgery and cannot formally demonstrate its presence at the exact time of the preceding CT. This temporal distinction is important when interpreting our findings. However, our study was not designed as a diagnostic-accuracy study and did not attempt to separately localize or segment recurrent tumor. The entire radiologically abnormal region was deliberately analyzed because necrotic, inflammatory, and potentially malignant components may be radiographically inseparable in this challenging clinical setting. The observed differences should therefore be interpreted as associations between quantitative features measured on preoperative CT and pathological classification at surgery, rather than direct imaging evidence of viable tumor at the index CT.

Finally, I fully agree that the statistical findings are hypothesis-generating. Three of 107 features showed nominal uncorrected associations, none remained significant after false-discovery-rate correction, and only three patients had associated recurrence [1]. Accordingly, no multivariable classifier was developed and no diagnostic performance was estimated. The sensitivity analyses were intended only to determine whether the direction of these exploratory associations depended on a single recurrence case, not to establish robustness or a validated recurrence-specific radiomic signature.

Our study therefore does not establish CT radiomics as a diagnostic tool for recurrence within ORN. Rather, it identifies preliminary quantitative imaging signals that warrant further investigation. Larger multicenter cohorts, closer temporal alignment between imaging and pathology, and detailed clinical and dosimetric characterization will be essential before clinical utility can be assessed.

I thank Topkan and colleagues for contributing to this important methodological discussion.

## CRediT authorship contribution statement

**Arnaud Beddok:** Writing – original draft.

## Funding

No specific funding was received for this correspondence.

## Declaration of competing interest

The authors declare that they have no known competing financial interests or personal relationships that could have appeared to influence the work reported in this paper.

## References

[1] Ozcan O., Laluc R., Carsin-Vue A., Assouly N., Brenet E., Da Silva Ribeiro Mota A. (2026). Radiomic analysis of pathologically confirmed mandibular osteoradionecrosis to identify cases with associated recurrence: a multicentric retrospective study. Phys Imaging Radiat Oncol.

[2] Peterson D.E., Koyfman S.A., Yarom N., Lynggaard C.D., Ismaila N., Forner L.E. (2024). Prevention and Management of Osteoradionecrosis in patients with head and neck Cancer treated with radiation therapy: ISOO-MASCC-ASCO guideline. J Clin Oncol Off J Am Soc Clin Oncol.

[3] Moreno A.C., Watson E.E., Humbert-Vidan L., Peterson D.E., van Dijk L.V., Urbano T.G. (2025). International expert-based consensus definition, classification criteria, and minimum data elements for osteoradionecrosis of the jaw: an interdisciplinary modified Delphi study. Int J Radiat Oncol Biol Phys.

